# How to Treat Hernias in Pregnant Women?

**DOI:** 10.7759/cureus.8959

**Published:** 2020-07-02

**Authors:** Nuaman A Danawar, Andrew Mekaiel, Sumit Raut, Ishani Reddy, Bilal Haider Malik

**Affiliations:** 1 General Surgery, California Institute of Behavioral Neurosciences and Psychology, Fairfield, USA; 2 Internal Medicine, California Institute of Behavioral Neurosciences and Psychology, Fairfield, USA; 3 Psychiatry and Behavioral Sciences, California Institute of Behavioral Neurosciences and Psychology, Fairfield, USA

**Keywords:** hernia, pregnant women, ventral hernia, umbilical hernia, laparoscopy, mesh, bariatric surgery

## Abstract

A hernia is a common surgical problem. Although hernias during pregnancy are uncommon, they can be challenging for both the surgeon and the patient if present. To date, there is no consensus in the medical community regarding the elective repair of hernias in pregnant women. The debate mainly concerns three areas: the timing, the approach, and the surgical technique. This study aims to offer a clear pathway in this field based on the best available data. In this study, we collected reviews written in English and published in PubMed from 2010 to 2020 (the exception being three articles that were published before 2010, which we retained since they contained relevant information). We used regular and Medical Subject Headings (MeSH) keywords. Two of the authors screened the collected studies to select the best articles that would fit our inclusion criteria for the review. The articles considered for this review can be classified into retrospective studies, case reports, and reviews. No randomized controlled trials were found.

The lack of an agreement about the treatment of ventral hernias in fertile women makes the decision to treat and the process challenging. The treatment significantly depends on two factors: the symptoms and the pregnancy status at diagnosis. If the hernia is incarcerated or strangulated at presentation, an emergency repair is obligatory. If the hernia is symptomatic, but not complicated, elective surgery should be offered. The timing of repair will depend on whether the patient is already pregnant or non-pregnant. In pregnant patients, if the hernia is small and asymptomatic, it may be better to delay the surgery until after delivery or after the last pregnancy. If the hernia is symptomatic and seems to affect the patient's quality of life, it may be better to postpone the repair until the second trimester or after delivery if complications do not occur. Internal herniation (IH) should be suspected as a cause of the abdominal pain in pregnant women who have undergone laparoscopic Roux-en-Y gastric bypass (LRYGB).

In pre-pregnancy patients, if the hernia is large and symptomatic, it may be better to do an elective repair and then wait for one or two years before the next pregnancy. However, if the hernia is small or minimally symptomatic, it may be better to hold the repair until after delivery or after the last pregnancy. Pregnancy may be considered a significant risk factor for hernia recurrence. The laparoscopic mesh repair should be offered whenever possible, whereas the open approach may be preferred in complicated cases. The suture repair may be suitable for both small hernias and in cases of gross contamination.

## Introduction and background

Hernias are a common surgical problem. Annually, about 20 million hernioplasties are performed globally [1]. According to the Danish Hernia Database, more than 130,000 inguinal hernia operations were performed in the last 11 years [2]. The hernia is a weakness in the musculofascial layer of the abdominal wall [3]. However, its occurrence is not confined to the abdominal wall. It can also occur in the perineum, diaphragm, and in the form of internal herniation (IH; the visceral herniation through mesenteric or omental defect). Many risk factors are involved in the development of a hernia. These factors include smoking, obesity, family history, hernia on the other side, male sex, older age, collagen disease, previous surgery, and pregnancy [1,4]. Pregnancy may be a significant risk factor for the hernia formation due to both hormonal changes and increased intra-abdominal pressure by the enlargement of the gravid uterus [4,5].

In the general population, the treatment of the hernias follows a clear strategy based on the international guidelines and recommendations [1]. The diagnosis is usually made by taking a detailed history and performing a thorough physical examination. Diagnostic images are used in rare cases. The ultrasound, CT, and MRI are the available radiological modalities that can be used in the diagnosis of atypical cases [2,6]. The treatment of hernias in this group is guided mainly by the symptoms at the time of diagnosis. Complicated hernias (incarcerated or strangulated ones) require emergency surgery. The laparoscopic mesh repair is the preferred approach for treating symptomatic hernias in both males and females. This approach is also preferred for asymptomatic hernias in females since asymptomatic hernias in females are more prone to complications compared to those in males [1,6]. Asymptomatic hernias in male patients may be treated by a watchful, waiting strategy [7].

In pregnant women, the exact prevalence of ventral hernias is unknown; however, it is estimated to be 0.08% for umbilical hernia and 0.12% for groin hernia [8,9]. Although hernias are uncommon during pregnancy, they can be challenging to treat when presented. Both the diagnosis and treatment of hernias in gravid women are challenging. Pain, nausea, vomiting, and leukocytosis are prevalent during pregnancy. Also, these findings are common in the case of complicated hernias as well. Diagnostic images may be mandatory when the diagnosis of hernias cannot be confirmed by physical examination. The risk of radiation exposure when using X-rays can be an issue; and CT and MRI are not available in all hospitals. These issues make the transabdominal ultrasound the modality of choice in gravid women. The use of ultrasound presents both pros and cons during pregnancy. On the one hand, it carries no radiation risk, is noninvasive, and can exclude other possible etiologies of the pain. However, it is operator-dependent and has reduced sensitivity in obese and pregnant patients. All these factors make the diagnosis of hernias challenging. The decision regarding an appropriate treatment option is also controversial due to the lack of high-quality clinical trials [9-11], and many questions remain unsolved: is the best time to repair hernia before, during, or after pregnancy? Is the laparoscopic approach better than an open approach? Is the mesh repair better than suturing alone?

In this study, we intended to find answers to these crucial questions and sketch a simple pathway for treating hernias in pregnant women. Our effort involved reviewing relevant articles in English published in PubMed from 2010 to 2020. This pathway will offer ways to streamline the treatment options, minimize complications, and maximize results. Additionally, this review may fill the gaps in the current research and help develop new research in this field.

## Review

Methods

This a traditional review article focusing on the treatment of the hernias in fertile women who are pregnant or planning to be pregnant in the future. The database used for this review article was PubMed. The keywords used for the data collection on PubMed are summarized in Tables 1, 2. The inclusion criterion was as follows: articles in the English language published in the last 10 years.

**Table 1:** Regular keywords used for literature search *Surgical prosthesis used in hernia repair

Keywords	Database	Number of results
Hernia	PubMed	93,281
Pregnant women	PubMed	114,636
Ventral hernia	PubMed	10,840
Umbilical hernia	PubMed	5,128
Laparoscopy	PubMed	111,990
Mesh*	PubMed	41,319
Bariatric surgery	PubMed	32,689

**Table 2:** MeSH keywords used for literature search MeSH: Medical Subject Headings

MeSH keywords	Database	Number of results
Hernia	PubMed	33
Pregnancy	PubMed	144
Surgery	PubMed	84

Results

The total number of results for various regular keywords were as follows: 93,281 for hernia, 114,636 for pregnant women, 10,840 for ventral hernia, 5,128 for umbilical hernia, 111,990 for laparoscopy, 41,319 for mesh, and 32,689 for bariatric surgery (BS), Similarly, the total number of results for MeSH keywords were: 33 for hernia, 144 for pregnancy, and 84 for surgery. After a thorough screening, a total of 48 research articles were finalized to draw a simple pathway for treating hernia before and during pregnancy, which is illustrated in this review article. However, some articles were excluded as they did not meet the inclusion criteria. We did not use any quality assessment tools for this review article.

Determining the best time for elective surgery

Currently, no clear protocol is available for treating ventral hernias during pregnancy. During pregnancy, both operative and nonoperative treatments are stressful. This is due to many reasons, such as dealing with two patients (mother and baby) at the same time, and the fact that elective surgery for uncomplicated hernias may carry unnecessary risks; however, emergency surgeries can turn hazardous if complications occurred during the nonoperative course.

The pregnancy status and the symptoms of hernia at the time of diagnosis are the essential factors for decision-making when treating hernias in fertile women. Complicated hernias at diagnosis (i.e., incarceration, strangulation, and bowel obstruction) require emergency surgery [9]. The elective repair requires more details and the process may differ based on if the patient is already pregnant or planning to be pregnant.

During pregnancy, if the hernia is small and asymptomatic, the watchful, waiting approach may be acceptable, and the repair can be delayed until after delivery or after the last pregnancy [12,5]. If the hernia is large and symptomatic, it may be better to postpone the repair to the second trimester, or until after delivery if the hernia remains stable. Hernia repair combined with a cesarean section (CS) involves a multifactorial decision-making process that varies from case to case, and this combination is not recommended routinely [9,10]; however, one study has revealed that this combination is an accepted practice [13]. Regarding groin hernia during pregnancy, it does not require routine repair [14]. Round ligament varicosities (RLV) are a common differential diagnosis of groin hernia, and they usually disappear within two to four weeks after delivery [15]. The recurrence rate of ventral hernias in women of reproductive age is about 12% [12]. Pregnancy following ventral hernia repair is associated with an increased recurrence rate. Hence, pregnancy may be considered as a risk factor for hernia recurrence [16].

Before pregnancy, if the hernia is small and asymptomatic, it does not require immediate repair and may be postponed until after delivery. If the hernia is large and symptomatic, it is better to do an elective repair and then wait for one to two years before the next pregnancy to give the patient enough time for hormonal and body-weight normalization [9].

Hence, in brief, the best time for treating ventral hernias in fertile women depends on pregnancy status and clinical presentation. Emergency cases may require immediate intervention, whereas elective processes can be delayed until postpartum or even after the last pregnancy. Semi-elective cases can be repaired in the second trimester of pregnancy, or one to two years before the planned pregnancy.

Nouh et al. conducted a systematic review study to evaluate the recurrence rate of ventral hernia in fertile women who underwent repair before pregnancy [12]. Oma et al. conducted another study with the same objective [10]. Both studies shared similarities in goal and patient groups but differed in terms of patient numbers and quality checks. The first study appears better as it was a meta-analysis with a significantly larger number of patients. However, both studies validated that pregnancy may be considered a significant risk factor for hernia recurrence. Hence, if possible, elective hernia repair should be delayed until after the last pregnancy.

Laparoscopic versus open approach

Once the issue of timing for the hernia repair is addressed, the next matter that should be dealt with is whether hernia should be repaired by an open or laparoscopic approach. Since the introduction of laparoscopic surgery in the 1980s, it has gained widespread acceptance among surgeons worldwide. The use of laparoscopic surgery in pregnant women was started much later due to technical and safety concerns. In the last 10 years, laparoscopy has become the standard approach for ventral hernia repair in the general population [6,17]. When comparing the open and laparoscopic approaches, the latter is associated with less hospital stay, less postoperative pain, early return to work, and fewer complications [17]. Choosing between a laparoscopic and open approach is a complex decision, and depends on many factors: patient age, sex, BMI, hernia size, comorbidities, surgeon's experience, previous surgery, available instruments, and patient preference. All these factors should be considered to achieve optimal outcomes [2,18]. The laparoscopic approach is generally considered superior to an open approach. Hence, it should be offered over an open approach for hernia repair in gravid women [19].

In gravid women, laparoscopic surgery may be safe in all trimesters. This element of safety can be maximized by a good understanding of the anatomical and physiological changes that occur in both mothers and babies during pregnancy. Moreover, establishing good coordination between an expert surgeon and anesthesiologist will also help. In an ideal scenario, the surgeon will keep the intra-abdominal pressure less than 12 mmHg, and the anesthesiologist will monitor the CO_2_. All these factors are essential to perform safe laparoscopic surgery on pregnant women. Furthermore, early diagnosis with prompt intervention is necessary for ensuring the safety of the patient during pregnancy [17,20,21]. Regarding the fetus outcome, recent studies have shown no difference between laparoscopic or open approach. Moreover, this may be affected by the underlying disease more than the surgery itself, and fetal acidosis occurring due to CO_2_ insufflation is still an unclear complication [19,21]. The laparoscopic repair of ventral hernia appears to be safe before and during pregnancy, without any significant impact on the pregnancy and delivery course. The laparoscopic approach also appears to be a safe process for treating BS complications during pregnancy, such as the laparoscopic management of IH after laparoscopic Roux-en-Y gastric bypass (LRYGB) [22,23]. The open approach may be offered in cases involving complicated hernia, thin patients, and large hernia size. The laparoscopic approach is preferred in females, obese, and recurrent hernias [2,18,24]. Consequently, good results of hernias repair in fertile women usually depend on good case selection. The laparoscopic approach should be offered whenever possible. The open approach should be reserved for complicated cases. Although the laparoscopic approach has many advantages over the open approach, these advantages should be balanced against the safety of the mother and the fetus.

Schoenmaeckers et al. conducted a study on eight patients to evaluate the effect of pregnancy following laparoscopic mesh repair of ventral hernia. They found that laparoscopic mesh repair in women who are planning to have more pregnancies is an acceptable therapeutic option, and causes no significant effects on pregnancy or delivery course. Five patients experienced tearing pain in the area of the repair in the last trimester of pregnancy. One patient had swelling on the repair site without signs of recurrence. The study did not mention the hazard of compartment syndrome during pregnancy due to the use of mesh in the hernia repair [23]. Lachman et al. reviewed 518 reported procedures during pregnancy. They revealed that laparoscopy during pregnancy seems to be safe when it is performed by experienced surgeons [20]. However, these two studies differ in many respects: the topic, the number of patients studied, and the timing of repair. The first study was done before pregnancy, but the second study involved women during the three trimesters of pregnancy. Both studies indicated that laparoscopy is a practical and safe approach for women of childbearing age before and during pregnancy.

Suture versus mesh repair

After deciding on the time and the approach for ventral hernia repair, a decision should be then made about choosing between a mesh or suture repair. Reviewing the available literature revealed that mesh repair might be better than suture repair alone. Both mesh and suture repair can cause pain in the third trimester of the next pregnancy [11]. Mesh repair might reduce the abdominal wall laxity during pregnancy [9].

Before pregnancy, the suture repair of small ventral hernias seems more suitable but may increase the risk of recurrence in the subsequent pregnancy. This risk of recurrence may be increased in the presence of diastasis recti; hence, mesh repair is recommended in this situation [9]. Mesh repair might reduce the recurrence risk but can cause chronic pain [25]. However, another study revealed that mesh repair was not associated with a reduction in the recurrence risk [26]. The mesh repair of ventral hernia and inguinal hernias appears to be a safe approach with no significant impact on pregnancy and labor course [15,24,27]. During pregnancy, suture repair may increase the risk of recurrence. Mesh repair may increase the risk of infection, especially in emergency cases [9]. The emergency mesh repair of the incarcerated hernias appears safe and accepted, without any significant postoperative complications [9,17]. Thus, in fertile women, the hernia repair with mesh is preferred and is the commonly followed and safer procedure, with no significant effect on pregnancy and labor course, while suture repair is usually reserved for emergency surgeries with the presence of contamination.

Jensen et al. conducted a systematic review that included 31 papers, of which 23 were case reports, to evaluate the treatment of abdominal wall hernias in fertile women before or during pregnancy. They found that the repair of the ventral hernia with suture or mesh might cause pain in the last trimester of the subsequent pregnancy [11]. Erling et al. conducted a study in Denmark on 441 women, who were planning for a subsequent pregnancy, to compare between mesh and suture repair. They found that mesh repair was associated with a reduced risk of recurrence but increased risk of chronic pain during the subsequent pregnancy [26]. Both studies were about the technique of hernia repair, but they differed in terms of the number of patients, quality checks, and the timing relative to the pregnancy. The study by Jensen et al. appears better since it was a systematic review, involved more patients, included patients before and during pregnancy, and used public databases (PubMed and Embase). The other study was a prospective cohort study with a fewer number of patients and used the national Denmark database only.

After reviewing the three essential steps for ventral hernia repair in fertile women, the current study proposed a short and simple pathway for achieving optimal outcomes for hernia treatment (Table 3).

**Table 3:** The proposed pathway for treating hernias in fertile women

Hernias in fertile women	Presentation	Timing	Symptoms	Treatment
	Emergency cases	Before and during pregnancy	Incarcerated, strangulated hernias	Requires emergency surgery. Open repair with mesh is preferred; suture is required if there is gross contamination
	Elective cases	Before pregnancy	Small, asymptomatic hernias	No repair for now; repair after childbirth or after last pregnancy
			Large, symptomatic hernias	Laparoscopic mesh repair, and then waiting for one to two years before a subsequent pregnancy
		During pregnancy	Small, asymptomatic hernias	Laparoscopic mesh repair after delivery or after the last pregnancy
			Large, symptomatic hernias	Laparoscopic mesh repair in the second trimester

Internal herniation after bariatric surgery

Obesity is a significant health problem worldwide these days. Among the available therapeutic options for obesity, BS appears to be the best weight-reduction therapy, with a better long-term outcome [28]. A considerable number of patients undergoing BS are females in their childbearing age, and most likely, they will become pregnant in the future. Thus, BS may have a serious impact on these patients during the subsequent pregnancy [29]. Our review of studies demonstrated that BS has positive effects on metabolic diseases and fertility; however, it may carry medical and surgical complications for mothers and babies, such as IH, bowel intussusception, bowel obstruction, gastric band slippage, bowel volvulus, gastro-jejunal bleeding, cholelithiasis, and maternal or fetal death [28]. To minimize these adverse effects, those who undergo BS are advised to avoid pregnancy for 12-24 months after BS [30]. However, currently, there is no consensus regarding the best time for conceiving after BS [28].

Among BS procedures, LRYGB has become the most popular procedure in the last 10 years. BS in fertile women increases the risk of abdominal surgery during the subsequent pregnancy, and LRYGB carries a specific risk of IH [31,32]. This risk should be kept in mind as a differential diagnosis of abdominal pain in pregnant patients who underwent BS [30]. The management of these patients is difficult as they usually present with minimal abdominal findings and apparently average laboratory results. However, any delay in the diagnosis and treatment can have a drastic impact [33]. Using the CT or MRI can increase diagnostic accuracy, and both these imaging modalities have comparable sensitivity and specificity [34]. If the diagnosis is still uncertain, laparoscopy can be offered as a diagnostic and therapeutic option [23,33]. IH can definitely be treated laparoscopically. The treatment consists of reducing hernia contents and closing the jejunal mesentery defect (Petersen's hernia). Postoperatively, the patients usually experience an uneventful pregnancy and delivery course [33]. Maternal and fetal mortality for this patient group was estimated at 2.5% and 7.5%, respectively [29]. Based on these studies, a high suspicion of IH should be maintained when managing pregnant patients complaining of abdominal pain with a surgical history of LRYGB. Early diagnosis is critical for proper management and optimal outcome.

Petrucciani et al. conducted a systematic study of 120 women with a previous history of BS who underwent emergency surgery during pregnancy. They found that LRYGB was the most common procedure and IH the most common complication. The surgical complications of a previous BS during pregnancy probably have critical consequences [29]. Stuart et al. also conducted a cohort study on a large number of patients to compare the risk of abdominal surgery during pregnancy between women who have undergone BS and obese women with BMIs of >35 without previous BS. They found that BS was associated with a higher risk of abdominal surgery during pregnancy [32]. Both the above-mentioned studies had a similar aim and topic; however, they differed in the number of patients, and the second study had a control group. The first study appears better as it was a systematic review that involved quality checks based on preferred reporting items for systematic reviews and meta-analyses (PRISMA) guidelines. However, both studies validated that BS in fertile women increases the risk of surgical complications during pregnancy.

Pregnancy following ventral hernia repair is considered a unique risk for recurrence, and this raises some important questions: why does recurrence happen in some patients and not in others? Are there any genetic, familial, or other factors that may have a direct impact on the recurrence? Is the use of biological meshes decreasing the chronic pain while simultaneously increasing the strength of repair? To answer these questions, we need further prospective studies. And these studies should also address other pertinent questions as well: do we need more clarity regarding the surgical type and approach or method of repair according to trimester? Does this stratification have any significance, or could it be that the question of trimester does not really matter? Are there any ethical concerns regarding prospective trials? Is the health education being provided to fertile obese women about reducing weight really decreasing hernia complications? Hopefully, future researches will provide more insight into these issues.

Limitations

This review has some limitations. We could not find any randomized controlled trials regarding the treatment of hernias in women of reproductive age. The only available studies were case reports, articles reviews, and retrospective studies. These studies were often sparse and small in size. Also, we found that the was a lack of proper follow-up with retrospective studies. Moreover, we may have missed out on some relevant information since we depended solely on the PubMed database and only included studies conducted in the last 10 years.

## Conclusions

Hernias are uncommon during pregnancy but can be challenging if present. Three important points should be considered when treating hernias in fertile women: the appropriate time for surgery, the approach, and the technique. Both the symptoms and pregnancy status determine the timing. Incarcerated and strangulated hernias inevitably need emergency surgery. In contrast, the repair of asymptomatic and small hernias either before or during pregnancy should be delayed until after delivery or after the last pregnancy, while symptomatic hernias should be repaired one to two years before planned pregnancy or in the second trimester of the current pregnancy. Regarding the approach and technique, the laparoscopic approach with mesh should be offered whenever possible. It should be kept in mind that IH could be a cause of the abdominal pain in gravid women who have undergone LRYGB.

We believe this study is clinically significant as it offered a short and straightforward pathway to treat hernias in fertile women. This pathway will help both obstetricians and surgeons to deal efficiently with this challenging subject. However, more studies of standardized design with a large number of patients are recommended to achieve a consensus on the treatment strategy. Also, we recommend treating these patients in specialized centers to improve the surgeon's experience and surgical outcomes, maintain proper follow-up, and collect more data about this field.
